# Supplementary material for: Prevalence, determinants, and association of overweight/obesity with non-communicable disease-related biomedical indicators: A cross-sectional study in schoolteachers in Kabul, Afghanistan
Source: PLOS Glob Public Health. 2023 Mar 7;3(3):e0001676. doi: 10.1371/journal.pgph.0001676 (PMC10021827; doi:10.1371/journal.pgph.0001676)
Supplement: S1 Codebook — (DOCX) [file pgph.0001676.s002.docx]

S1 Codebook: Codebook_NCD study_overweight_obesity.docx

| Variable | Label | Values |
| --- | --- | --- |
| sex | Sex of the participant | 1 Male  2 Female |
| age10year | Age in years (10-year category) | 0 18 – 30  1 31 – 40  2 41 – 50  3 ≥51 |
| education | Education attainment | 1 12^th^ grade (high school) graduate  2 14^th^ grade (2-year college) graduate  3 College/university graduate or higher |
| marital_status | Marital status | 0 Never married  1 Currently married |
| work_experience | Teaching experience in years | 0 <10  1 10-20  2 ≥21 |
| income | Monthly household income in Afghanis | 0 Less than or equal to 10,000 Afs  1 More than 10,000 but less than 20,000 Afs  2 More than 20,000 |
| phys_activity | Physical exercise/walking (per day) | 0 <1 hour  1 ≥1 hour |
| fruit_veg_intake | Consumption of fruits/vegetables (per week) | 0 <4 times  1 ≥4 times |
| tobacco_use | Tobacco use (cigarette, smokeless tobacco, water pipe) | 0 No  1 Yes |
| weight_cont | Body weight | (data are in kilograms) |
| height_cont | Body height | (data are in centimeters) |
| bmi_cont | Body mass index | (data are in kg/m^2^) |
| overweight_or_obese | Overweight/obesity status | 0 Non-obese (BMI<25.0 kg/m^2^)  1 Overweight/obese (BMI≥25.0 kg/m^2^) |
| bmi_3cats | Body mass index in 3 categories | 0 underweight (BMI<18.5 kg/m^2^)  1 normal weight (BMI=18.5-25 kg/m^2^)  2 overweight/obese (BMI≥25.0 kg/m^2^) |
| systolic1 | Systolic blood pressure (first measurement) | (data are in mmHg) |
| diastolic1 | Diastolic blood pressure (first measurement) | (data are in mmHg) |
| systolic2 | Systolic blood pressure (second measurement) | (data are in mmHg) |
| diastolic2 | Diastolic blood pressure (second measurement) | (data are in mmHg) |
| systolic_avg | Average systolic blood pressure | (data are in mmHg) |
| diastolic_avg | Average diastolic blood pressure | (data are in mmHg) |
| blood_pressure_cat | Blood pressure (in mmHg) | 0 <130/85  1 ≥130/85 |
| drug_htn | Took any drugs for raised blood pressure in the past two weeks? | 1 Yes  2 No |
| hba1c_cont | Glycated hemoglobin level (in %) | (data are in %) |
| hba1c_recode | Glycated hemoglobin categories | 0 <5.5  1 ≥5.5 |
| drug_diabetes | Took any drugs for diabetes in the past two weeks? | 1 Yes  2 No |
| cholesterol_cont | Total cholesterol level | (data are in mg/dL) |
| cholesterol_recode | Total cholesterol categories | 0 <200  1 ≥200 |
| drug_highchol | Took any drugs for raised blood cholesterol in the past two weeks? | 1 Yes  2 No |
| triglycerides | Triglycerides level | Data are in mg/dL |
| triglyceride_recode | Triglycerides level in categories | 0 <150  1 ≥150 |
| hdl | High density lipoprotein cholesterol level | Data are in mg/dL |
| hdl_recode | HDL level categories | 0 ≥40  1 <40 |
| ldl | Low density lipoprotein cholesterol level | Data are in mg/dL |
| ldl_recode | LDL level categories | 0 <100  1 ≥100 |
| comorbid_sum | Sum of comorbidities (hypertension, elevated HbA1c, high total cholesterol, high LDL, low-HDL, and high  triglycerides. | Number of comorbidities |
| comorbid_recode | Comorbidities recoded | 0 less than three  1 ≥3 or more |
