# Supplementary material for: Prevalence, determinants, and association of overweight/obesity with non-communicable disease-related biomedical indicators: A cross-sectional study in schoolteachers in Kabul, Afghanistan
Source: PLOS Glob Public Health. 2023 Mar 7;3(3):e0001676. doi: 10.1371/journal.pgph.0001676 (PMC10021827; doi:10.1371/journal.pgph.0001676)
Supplement: Attachment — Submitted filename: PGPH-D-22-01171-R1-Response_to_Reviewers-1029.docx [file pgph.0001676.s003.docx]

Response to reviewers

Manuscript (PGPH-D-22-01171): Prevalence, determinants, and association of overweight/obesity with non-communicable disease-related biomedical indicators: a cross-sectional study in schoolteachers in Kabul, Afghanistan

The authors would like to thank all reviewers for providing constructive suggestions and comments on our manuscript. We have addressed reviewers’ comments and suggestions in the manuscript and included a point-by-point response to each comment. According to the reviewer’s suggestion, the authors performed the analysis using the Poisson regression model. The results for the relationship with overweight/obesity are generally consistent with the earlier results. The Methods, and Results sections and Tables 3-5 were amended according to the re-calculated statistical analysis. We believe that the revised manuscript is much improved based on the feedback from the reviewers.

**Editor comments**

1. Please provide your detailed Financial Disclosure statement. This is published with the article. It must therefore be completed in full sentences and contain the exact wording you wish to be published.

Response: The authors appreciate the editor’s suggestion. The funding source is elaborated in the “Funding” section (Page 17, lines 329-330).

2. State the initials, alongside each funding source, of each author to receive each grant.

Response: The grant recipient was elaborated by the initials.

3. We have noticed that you have uploaded Supporting Information files, but you have not included a list of legends. Please add a full list of legends for your Supporting Information files after the references list.

Response: The legend for the supporting information files is added after the reference list (Page 22, lines 493-497).

**Reviewer #1:**

I congratulate the authors for submitting this manuscript to PLoS Global Public Health. There is a dearth of updated information on the prevalence of NCD-related biomedical indicators in Afghanistan. This manuscript fills a critical knowledge gap.

The manuscript, however, needs substantial revision to be considered for publication. I focused on methodological issues, which are listed under the major comments. I also listed some minor comments for the authors’ consideration.

Major comments:
1. The prevalence of overweight/obesity and NCD-related biomedical indicators was found to be high (p.8, para 4). In the situation where the binary outcome is common, i.e., the prevalence is greater than 10%, the odds ratio (OR) estimated from binary logistic regression overestimates or jeopardizes the true measure of association. In such cases, a prevalence ratio (PR) instead of OR is a suitable option. Authors should consider using Poisson regression models with robust variance, log-binomial, or other advanced methods for calculating the adjusted PR.

Response: The authors appreciate the reviewer’s suggestion. The authors have re-performed the analysis using the Poisson regression model with robust variance to report an adjusted prevalence ratio. The results in Tables 3-5 are replaced with the Poisson regression analysis. The results of statistically significant relationships are consistent with the previous analysis. The values of prevalence ratios are slightly smaller than the values of odds ratios. The abstract and the main text (Methods and Results sections) were amended according to the updated numerical results obtained by the Poisson regression analysis. (Page 2, line 27, lines 32-36; Page 8, lines 149-151; Page 9, lines 161-168; Pages 10-11, lines 190-229; Tables 3-5)

2. Both the sub-sections in p.9 (both (this and above) are results from logistic regression analyses. Please consider revising the titles (“Factors associated with overweight/obesity” and “Results of logistic regression analysis”) to make those self-explanatory. Also, please consider explaining it in the “statistics analysis” section so that readers can understand which dependent variable(s) was used for which set of analyses.

Response: According to the reviewer’s suggestion, the subheadings were amended. Dependent variables included in the analysis and model adjustment were elaborated on in the “statistical analysis” section. (Page 10, lines 189 and 194; Page 8, lines 153-155, lines 156-157)

3. It is unclear whether the authors checked multi-collinearity, interaction, or effect modification in the data (for other than sex).

Response: According to the reviewer’s suggestion, multicollinearity, effect modification, and assumptions of the Poisson regression model were checked prior to fitting the model. Description of the tests and parameters were elaborated on in the Methods and Results sections. (Page 9, lines 162-167; Page 11, lines 227-229)

Minor comments:
1. Apart from the abstract, line numbers for the manuscript texts were not provided. This would be useful for making specific comments.

Response: The authors apologize for missing line numbers on some pages in the earlier manuscript. According to the reviewer’s comment, line numbers were included throughout the manuscript.

2. Both the full title and the short title should consider specifying Kabul, Afghanistan, since the data are from 22 municipal districts of Kabul only.

Response: According to the reviewer’s comment, “Kabul” was specified in the titles. (Page 1, lines 1-6)

3. The authors should consider revising the statement (“This was the first study to be conducted on an Afghan population …”) on p.13, para 3 to acknowledge the existing works on similar topics in Afghanistan (viz., Pengpid & Peltzer (2021) doi: 10.1186/s41043-021-00251-0; Saeed (2013) doi: 10.5195/cajgh.2013.69; Saeed et al. (2020) doi: 10.26719/emhj.20.005; Sahrai et al. (2022) doi: 10.1007/s44197-021-00026-0)

Response: According to the reviewer’s comment, the statement was revised, and recently published works on overweight and obesity in Afghanistan were added in the Introduction and Discussion sections. (Page 4, lines 65-67; Page 12, lines 237-239; Page 13, lines 257-260; Page 15, lines 311-312)

4. In Tables 1 and 2, consider replacing “Non-obese” with “Non-overweight/obese.”

Response: According to the reviewer’s comment, expressions in the column headings in Tables 1 and 2 and the main text were corrected. (Page 10, lines 197 and 203; Page 11, lines 224 and 226; Page 13, line 253; Page 23, Table 1; Page 24, Table 2)

**Reviewer #2**

This cross-sectional study aimed to determine the prevalence, determinants, and association of overweight/obesity with NCD-related biomedical indicators among schoolteachers in Afghanistan. Furthermore, the authors wanted to emphasize the role overweight/obesity in the development of NCDs among school teachers in Afghanistan. This study present evidence-based findings that call for more robust future studies as well as advocate for public health policy to improve adult’s health in Afghanistan. Here, I highlight some of minor and major considerations to be considered prior to publication.

Minor comments
1. Title: please rewrite the title to read as “Prevalence, determinants, and association of overweight/obesity with noncommunicable diseases-related biomedical indicators: a cross-sectional study in schoolteachers in Afghanistan”.

Response: According to the reviewer’s suggestion, the title was amended.

2. Introduction
Please add more information about the burden of NCDs and its related risk factors specifically in general population in Afghanistan.

Response: According to the reviewer’s comment, information on the NCD burden and risk factors in the general population in Afghanistan was addressed. (Page 4, lines 54-67)

3. Methods
Please elaborate more on how the random selection was implemented.

Response: According to the reviewer’s comment, the selection process of the study sample was elaborated on in the Methods section. (Page 6, lines 108-113)

4. For the six measured dependents variables, please elaborate more on which bases those specific cut-off values were used to categories those variables into dichotomous variables.

Response: According to the reviewer’s comment, the application of specific cut-off values was elaborated, and sentences were amended in the Methods section. (Page 8, lines 138-144)

5. Statistical analysis: please consider writing that ‘to investigate the effect modification of sex on the association between exploratory variable and measured outcomes, sex-stratified analysis was….’

Response: According to the reviewer’s advice, the sentence was amended. (Page 8, lines 155-156)

6. Results
Please elaborate more on how many were randomly selected and how many consented to participate in this study to reflect the response rate.

Response: According to the reviewer’s comments, a description of the selection of the study participants was added in the Methods section. (Page 6, lines 108-113)

7. Before addressing the prevalence of overweight/obesity and the deference in that by the measured variables, please provide some descriptive on the collected characteristics of the study population.

Response: According to the reviewer’s suggestion, the characteristics of the study population were elaborated. (Page 9, lines 177-180)

8. Please replace ‘probability’ with ‘odds’. Probability reelects the risk while odds reflect the likelihood.

Response: According to the reviewer’s comment, the appropriate term was used throughout the manuscript to reflect the likelihood based on the re-performed analysis.

9. Table 2.
I suggest for the sub-categories of the measured biochemical indicators to be written as ‘elevated / normal; whenever applicable. Cut-off values could be provided in brackets for each sub-category.

Response: According to the reviewer’s comment, “elevated/normal” was used and column headings were amended. (Tables 2, 4, and 5).

10. The column labeled n(%) do not sum up to 600. Please clarify more why there are some missing values in almost all of the measured biomedical indicators. Providing a flow chart presenting number of study participants tested for each of the measured NCD-related biomedical indicator would help.

Response: In the analysis of NCD-related biomedical indicators, participants who were taking medication for hypertension, diabetes, and hyperlipidemia were excluded in order to avoid potential bias. The sentence in the Methods section was amended to reflect the sample size included in the analysis. The footnotes in Tables 2, 4, and 5 indicates the number of excluded participants in each set of analysis. (Pages 8-9, lines 158-161)

Overall, the paper reads very well. Minor English editing might be needed. Considering all of the above comments would improve the readability and understandability of the paper when it published.

Response: Thank you very much. We have revised the manuscript to improve readability.
