# Supplementary material for: Prevalence, determinants, and association of overweight/obesity with non-communicable disease-related biomedical indicators: A cross-sectional study in schoolteachers in Kabul, Afghanistan
Source: PLOS Glob Public Health. 2023 Mar 7;3(3):e0001676. doi: 10.1371/journal.pgph.0001676 (PMC10021827; doi:10.1371/journal.pgph.0001676)
Supplement: Attachment — Submitted filename: Response to reviewers-R2.docx [file pgph.0001676.s004.docx]

Response to Reviewers

PGPH-D-22-01171-R2 - Prevalence, determinants, and association of overweight/obesity with non-communicable disease-related biomedical indicators: a cross-sectional study in schoolteachers in Kabul, Afghanistan

**Reviewer #1**

All comments have been addressed

Response: Thank you very much.

**Reviewer #3**

The paper focuses on prevalence of NCDRF among teachers and reports a higher prevalence.

1. The choice of teachers as a specific group is not well justified. They are definitely of higher SES. What factors in obesity causal pathways are different in teachers. A better justification is required in the introduction.

Response: According to the reviewer’s comment, a sentence was added to justify the choice of schoolteachers for this study (Page 4, lines 63-65).

2. Introduction is too long and can be shortened to half.

Response: According to the reviewer’s comment, the introduction section was revised and shortened.

3. With such high prevalence, is it justified to say the overweight and obesity are "emerging" public health concerns.

Response: According to the reviewer’s comment, the sentence was amended (Page 4, line 45).

4. Selection of participants for the survey raises serious issues of bias - it seems to be by referral of Principals of the school and not really a random sample.

Response: Thank you for the comment. The procedure for the selection of the study sample was elaborated, and a sentence was added in the Discussion section on the limitation (Page 6, lines 99-100; page 15, lines 323-325).

5. Why was a poisson regression model used? Will not obesity be a dependent variable in analysis and not explanatory variable?

Response: The authors appreciate the reviewer’s comment. The application of Poisson regression model was based on the high prevalence (>10%) of the six binary outcome variables. Evidence was provided to support the selection of the model for our analysis (Page 7, lines 139-140).

6. Not clear why people on treatment for HT/DM/Hyperlipidemia were excluded from analysis. This could have introduced a bias, though the authors justify is that this was done to avoid bias.

Response: According to the reviewer’s comment, the reason for the exclusion of the participants under medication was elaborated (Page 8, lines 149-151).

7. The whole issue of female gender as an effect modifier is not well addressed.

Response: According to the reviewer’s comment, sentences were added in the discussion section to elaborate on the issue of sex differences (Pages 13, lines 279-286).

8. Association of obesity with biomedical indicators is well known and does not really add anything.

Response: According to the reviewer’s comment, a sentence was added in the introduction section to address the significance of the study (Page 5, lines 79-83).

9. Table 4 is just too much analysis without any value addition, just because data are available.

Response: Thank you for the comment. The association between overweight/obesity and NCD-related biomedical indicators is presented in Table 4, which is one of the main tables to present the important findings of the paper to support the hypothesis.

**Reviewer #4.**

The authors are to be congratulated on an important and useful paper. I have some comments/suggestions that could be used to improve the paper:

1. Authors mentioned:

‘In the total sample (n=600), 58.2% were classified as 180 overweight/obese’.

Question: Is this over all prevalence? Authors need to show their reads separate analysis for mean/prevalence of overweight and obesity for males and females.

Response: The authors appreciate the reviewer’s comment. The overall prevalence of overweight/obesity in this study was 58.2% representing 349/600 participants. The distribution of overweight/obesity is also calculated for males and females in Table 1. The sentence was revised to clarify the differences in the prevalence of overweight/obesity according to the sex of the participants (Page 9, lines 173-174).

2. Authors mentioned:

‘The study team worked with a joint committee of the Afghanistan Ministry of Public Health (MoPH) and the Ministry of Education (MoE) and prepared listwise information on all 210 primary, middle, and high public schools across 22 municipal districts of Kabul. Based on the formal invitation letter from the MoPH and MoE, the principals of individual schools selected and introduced two to three schoolteachers who met the eligibility criteria and consented to participate in the study’.

Question: To my opinion, the methodology of the formation of the study, how the selection was done?

Response: According to the reviewer’s comment, the sentences were amended to clarify the selection of the participants (Page 6, lines 99-100)

3. Authors mentioned in the discussion (Sex differences in the prevalence of overweight/obesity)

Question: Is there a specific reason for Afghanistan cultural/social why women are more overweight/obese compared to men.

Response: According to the reviewer’s comment, sentences were added to further explain the cultural and traditional aspects in the Afghanistan context (Page 14, lines 296-300).

4. Authors did not explain whether all the participants underwent questionaries, anthropometric measurements and blood biochemistry tests?

Response: According to the reviewer’s comment, the sentence was revised (Page 6, lines 111-113).
