# Supplementary material for: Prevalence, determinants, and association of overweight/obesity with non-communicable disease-related biomedical indicators: A cross-sectional study in schoolteachers in Kabul, Afghanistan
Source: PLOS Glob Public Health. 2023 Mar 7;3(3):e0001676. doi: 10.1371/journal.pgph.0001676 (PMC10021827; doi:10.1371/journal.pgph.0001676)
Supplement: Attachment — Submitted filename: Response to reviewers-R3-20230207.docx [file pgph.0001676.s005.docx]

Response to reviewers-R3

PGPH-D-22-01171-R3 - Prevalence, determinants, and association of overweight/obesity with non-communicable disease-related biomedical indicators: a cross-sectional study in schoolteachers in Kabul, Afghanistan

**Reviewer #3**

One needs to encourage publications from Afghanistan. However, the paper still needs to be improved. Issues raised have not been adequately addressed.

Response: According to the reviewer’s comment, the manuscript has been extensively revised to address the issues raised. The following is a more thorough explanation of each point:

1. It is clear that the sample has a serious selection bias that cannot be corrected now. But they can compare the characteristics of the sample with the teacher population if Afghanistan (if available) For example, 69% were women in the sample, does it hold true for all Afghanistan teachers. Using this estimate to make generalization about all teachers in Afghanistan is inappropriate.

Response: We appreciate the reviewer’s comment on the characteristics of the sample. According to the official figures from the Afghanistan Ministry of Education, out of 29,876 schoolteachers in Kabul city, 20,021 (67%) are female, which is in line with the composition of the sample selected for our study. A sentence has been added on the selected sample under ‘study design and setting’ in the Methods section (Page 6, lines 108-110).

2. Exclusion of HT/DM on treatment is also not acceptable as it misclassifies individuals. It is not clear why a reanalysis cannot be done with the appropriate classification.

Response: According to the reviewer’s comment, the revised analyses include all participants regardless of reporting history of drug treatment for hypertension, diabetes, and dyslipidemia. The results of both analyses (with and without including participants under treatment) were similar and did not alter the relationship between the explanatory variable and outcome measures. Changes in the p-value for HDL-C and overweight/obesity status (0.019 to 0.009) (Table 2), the p-value for sex and hypertension (significant to insignificant) (Table 4), and p-value for overweight/obesity and elevated total cholesterol among female teachers (insignificant to significant) (Table 5) are a few to mention. Therefore, the analyses, including all participants, were considered for this paper, and the manuscript was thoroughly revised according to updated results (Tables 2, 4, and 5).

3. Gender issues does need better exploration. In Discussion, they have just repeated the results and said it needs exploration. If this paper has to add value it has to be in these kinds of issues.

Response: According to the reviewer’s comment, the gender issue was further discussed in the Discussion section, and the sentences were revised (Pages 13 and 14, lines 283-296; Page 13, lines 300-302).

4. Finding relationship between different metabolic variables is hardly of any significance for Afghanistan or any other country. Table 4 adds little value.

Response: Thank you for your suggestion. In Table 4, the main results of the association between overweight/obesity and multiple NCD-related biomedical indicators are presented. These indicators were measured objectively among a large sample of teachers and investigating their association with weight status is of vital importance in public health practice and policy research. The significance of this study has been further elaborated on in the Introduction section (Page 5, lines 70-75; Page 5, lines 85-87)

5. Discussion also has to focus on how to address this problem in schoolteachers as a specific risk group especially school based interventions.

Response: According to the reviewer’s comment, sentences were added in the Discussion section to address specific interventions (Page 15, lines 324-330).

**Reviewer #5**

The authors have reported a cross sectional study of obesity and associated NCD risk factors among school teachers in Afghanistan.

1. Are there any reasons to believe that obesity and other NCD risk factors are not likely to be related among teachers? Why is studying the teacher population important? Are there occupationally any unique risk factors that do not operate among other occupational groups or the general population? Such justifications will help strengthen the paper.

Response: According to the reviewer’s comments, the issue discussed in the Introduction section was further elaborated (Page 5, lines 70-75; Page 5, lines 85-87).

2. The sampling method has involved the schools selecting and volunteering a few teachers meeting the eligibility criteria from each school. Such volunteer-based sampling, that too done by the school administration is likely to be biased.

Response: According to the reviewer’s comment, the sentence indicating sample selection has been amended in the Methods, and the limitation of such sampling has been acknowledged and elaborated on in the limitation section (Page 6, lines 104-110; Page 16, lines 342-345).
